# Social participation of women with breast cancer compared to the general population 5 years after primary surgery—what role do medical data and cancer-related complaints play?

**DOI:** 10.1007/s00520-024-08695-w

**Published:** 2024-08-02

**Authors:** Stefanie Sperlich, Dorothee Noeres, Sophia Holthausen-Markou, Tjoung-Won Park-Simon, Eranda Sahiti, Siegfried Geyer

**Affiliations:** 1 Hannover Medical School, Department of Medical Sociology, Hannover, Germany; 2 Hannover Medical School, Gynaecological Psychosomatics and Psychooncology Clinic for Gynaecology and Obstetrics, Hannover, Germany; 3 Hannover Medical School, Gynecology Department, Hannover, Germany

**Keywords:** Social participation, Primary surgery, Breast cancer

## Abstract

**Purpose:**

This study analyzes levels of social participation in patients with breast cancer on average 5 years following primary surgery as compared to women in the general population. In addition, the role of breast cancer-related complaints and medical data as possible influencing factors on levels of patients’ social participation is investigated.

**Methods:**

A total of *n* = 454 patients after primary surgery (t0) were recruited for a third follow-up study, and *n* = 372 completed this survey (t3), corresponding to a response rate of 82.2%. For measuring breast cancer-related complaints, participants completed a written questionnaire. Social participation was measured by a questionnaire on different leisure activities that was taken from the Socio-Economic Panel Study. Medical information was extracted from medical reports at t0. A principal component analysis was carried out to identify different dimensions of social participation. Chi^2^-tests and logistic regression analyses were applied to analyze social participation as compared to the general population and the role of possible medical and diagnosis-related influencing factors thereby.

**Results:**

Compared to the general population, patients show lower levels of social participation in the domains “socio-cultural participation” and “participation in institutions,” while no significant differences for “social participation in the private sphere” and “social participation via social media” were found. Psychological symptoms, pain, and a history of mastectomy were most strongly associated with restrictions in social participation.

**Conclusions:**

Our study suggests that social withdrawal may happen due to disease-related symptoms, preventing some breast cancer patients from participating fully in society. Cancer-related follow-ups should address this issue and support patients’ reintegration into society through appropriate therapeutic interventions.

**Supplementary Information:**

The online version contains supplementary material available at 10.1007/s00520-024-08695-w.

## Introduction

Breast cancer is the most common cancer in women, with around 70,000 new cases every year in Germany and a lifetime prevalence of 12.5% [1]. Due to advances in early diagnosis and more effective treatments, breast cancer mortality rates have been declining steadily, with women between 55 and 69 years of age benefitting most from increased survival [1]. As a consequence, breast cancer becomes a chronic condition rather than a life-threatening illness [2]. Issues relating to the long-term effects of breast cancer and health-related quality of life (HRQoL), which encompasses the physical, mental and social dimensions of health, have become increasingly important for medical care [3–5]. Previous studies have shown that physical, social, and emotional function is impaired in breast cancer survivors, leading to a poorer HRQoL [6–9]. A recent review found that HRQoL in breast cancer patients improved significantly in recent years since interventions such as physical activity and psychosocial interventions proved to be effective. However, the authors stated that symptoms like pain, lymphedema, and worry caused by different treatment modalities are still relevant and need more serious attention and further consideration in order to improve HRQoL in breast cancer patients [3].

Social participation, defined as a “ person’s involvement in social activities that provide social interactions within his/her community or society” [10], is linked with better functionality and HRQoL [11–16]. Symptoms associated with a breast cancer diagnosis and the side effects of treatment such as anxiety, depression, fatigue and pain can have a significant impact on patients’ functional health and lead to restrictions in their social activities [3, 17]. The International Classification of Functioning, Disability and Health (ICF) proposed by the World Health Organisatzion conceptualizes a person’s level of functional health as a dynamic interaction between her or his health conditions, environmental factors, and personal factors. The ICF encompasses the components “activities” and “participation” as crucial dimensions of functional health that can be affected by health conditions like breast cancer [18]. According to the ICF nomenclature, “activity” is the performance of a task or an action by a person while “participation” is being involved in a life situation. For example, social participation in the ICF implies to maintain internal relationships to family, relatives, acquaintances, and friends (code d7) and to participate in community, social and civic life, e.g., by going to church, taking part in cultural activities, leisure activities or going on holiday (code d9). The core goal of rehabilitation is to enable social participation and return to normal life and society [11].

As Zhu et al. pointed out, research on social participation in breast cancer patients is still in its infancy, and most studies have been cross-sectional so far [11]. In particular, there is a lack of studies on the long-term effects of breast cancer on social participation.

The aim of this study is to take a step towards closing this research gap and investigates the effects of breast cancer and its treatment on social participation of patients on average 5 years after first surgery. In more detail, we first analyze social activities in different dimensions on daily living as compared to the general population of women in Germany. In a next step, we investigate the role of diagnosis-related complaints and medical data as potential influencing factors on the levels of social participation.

## Methods

### Data source

#### Breast cancer patients

The study population is based on participants of a multicenter longitudinal study on return to work after breast cancer. Between November 2016 and September 2018, a total of *n* = 562 employed breast cancer patients who had received primary surgery were invited to participate in the study. Patients were considered eligible if they had a diagnosis of invasive primary breast cancer, were not older than 63 years, working at the time of diagnosis, and did not exclusively have a ductal carcinoma in situ (DCIS) [19]. The study was performed in accordance with the ethical standards as laid down in the 1964 Declaration of Helsinki and its later amendments or comparable ethical standards. It was approved by the ethics committee of Hanover Medical School under the number 2973–2015.

The recruitment in Lower Saxony took place in 10 certified breast cancer centers and additionally at one gynaecologist’s practice cooperating with two certified breast cancer centers. Baseline data (t0) on *n* = 454 employed breast cancer patients were collected in the first weeks after primary surgery, corresponding to a response rate of 80.7%. For the follow-up assessments 6 months (t1), 12 months (t2), and 4–6 years (mean: 5.2 years) (t3) after primary surgery, patients were sent written questionnaires to their homes. The last follow-up (t3), which represents the data basis of this study, focused on the social participation of breast cancer patients on average 5 years after the primary surgery. A total of *n* = 372 women took part in this final survey, which was conducted between November 2022 and October 2023, corresponding to a response rate of 82.2% in relation to the initial sample (t0). This study is based on *n* = 346, which corresponds to the number of responses received by August 2023. The sample characteristics are displayed in Table 1. The number of missing values on the variables included varied between *n* = 0 and *n* = 45. Respondents with missing information were excluded.

**Table 1:** Sample characteristics of breast cancer patients (*n* = 346)

	*n*	%
Age group		
20–39 yrs	14	4.1
40–49 yrs	45	13.1
50–59 yrs	174	50.3
60–69 yrs	113	32.6
missing	0	
Educational level		
low (up to 9 yrs. of schooling)	44	12.8
intermediate (10 yrs. of schooling)	136	41.1
high (12 to13 yrs. of schooling)	155	46.1
missing	11	
Household net income		
up to 1749 €	52	17.4
1750 to 3999 €	166	55.5
4000 + €	81	27.1
missing	47	
Family status		
married	223	65.1
single	43	12.5
divorced /separated	66	19.2
widowed	11	3.2
missing	4	
Migration background^1^		
yes	61	18.2
no	274	81.8
missing	11	
Tumor size		
≤ 2 cm	211	61.2
2 to 5 cm	109	31.5
> 5 cm	25	7.3
missing	1	
Chemotherapy received		
yes	188	54.3
no	158	45.7
missing	0	
Mastectomy		
yes	79	22.8
no	263	77.2
missing	4	
Axiallary lymph node status		
0 (none node contain cancer)	273	79.1
1	58	16.8
2 +	14	4.1
missing	1	
Grading		
1 (cancer cells well differentiated)	43	12.6
2 (cancer cells moderately differentiated)	179	52.5
3 (cancer cells poor differentiated)	119	34.9
missing	5	
At t3 on anti-hormone therapy		
yes	167	48.7
no	176	51.3
missing	3	

^1^ migration background = at least one parent was not born in Germany

### General population (GSOEP-study)

We used the data from the German Socio-Economic Panel study (GSOEP V.31) to compare the social participation of breast cancer patients with the general population. The GSOEP is a representative annual survey of German individuals aged 18 and older in private households, conducted by the German Institute for Economic Research. Every year, approximately 30,000 people in 22,000 households are interviewed for the SOEP study. The SOEP is funded by the Federal Ministry of Education and Research (BMBF) and the German federal states [20]. The central survey instrument for this study is an individual questionnaire on people’s social activities in their leisure time, covering 20 different activities, which each adult household member is supposed to answer. We used the data from the 2019 survey year, which was still unaffected by the coronavirus pandemic. Further information on GSOEP can be obtained from Goebel et al. [20]. For comparison, the sample of the breast cancer patients and the GSOEP sample were parallelized with regard to key socio-demographic variables, i.e., each patient was matched with 3 women from the GSOEP (*n* = 1035) who were similar in terms of age and education and who did not have a cancer diagnosis. This procedure should ensure that the social backgrounds of cases and controls were comparable.

### Measures

#### Breast cancer-related complaints

**Fig. 1:**
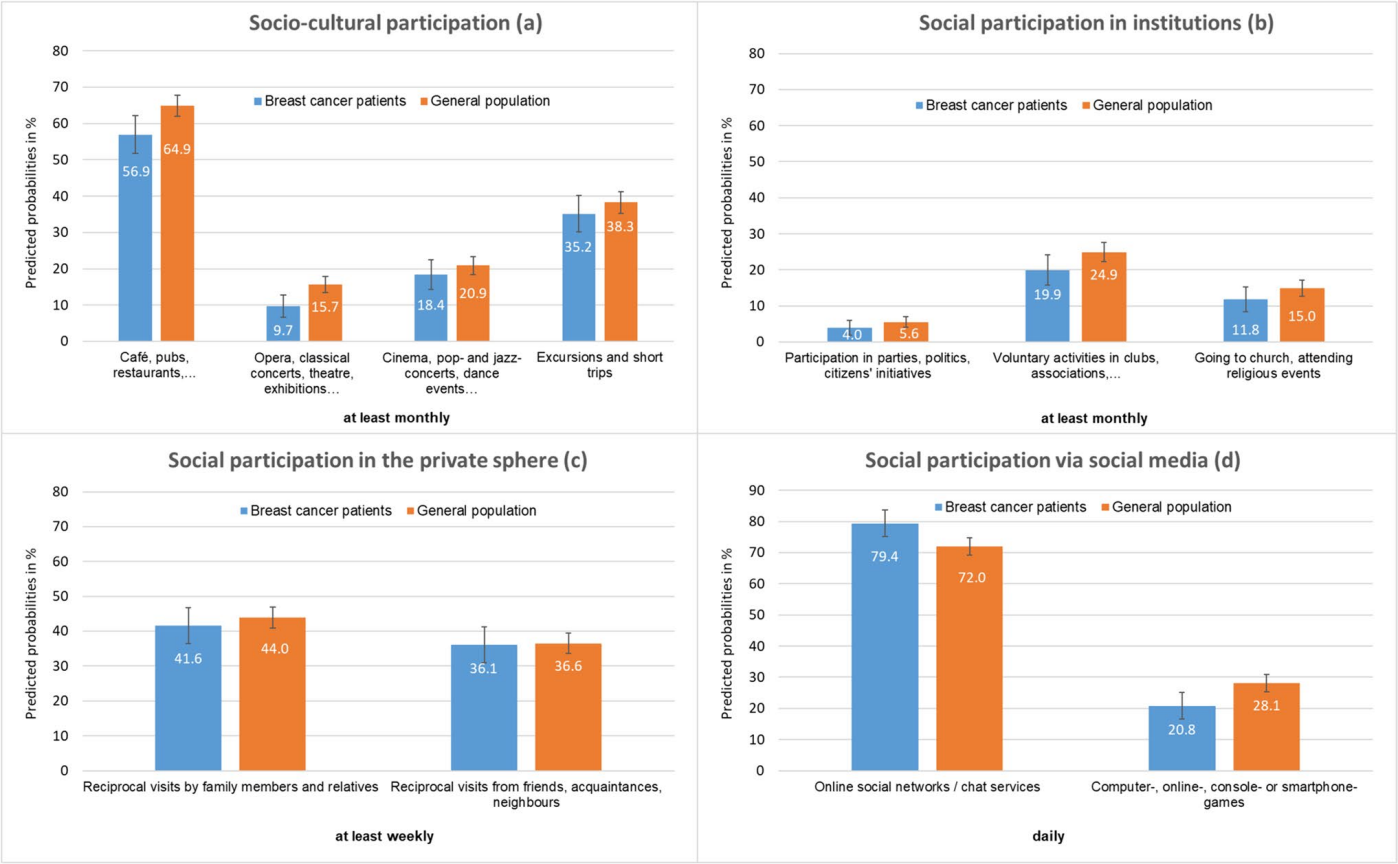
Adjusted prevalence (predicted probabilities) of social participation in four dimensions (**a** to **d**) among breast cancer patients (*n* = 346) as compared to women of the general population (*n* = 1035)

Patients were given a list of 18 different diagnosis-related complaints that are typically associated with breast cancer and can occur even years after diagnosis [21]. This list was compiled on the basis of the respective literature [22], supplemented by selected freetext answers of the patients of our study one year after primary surgery (t2) as well as based on the medical expertise of the gynaecologists in our working group at Hannover Medical School. For measuring the diagnosis-related complaints, the patients were asked the following question as part of a written questionnaire at t3: “Do you currently have any complaints as a result of breast cancer or as a result of breast cancer treatment that affect your everyday life? Please check for each answer option whether it applies.” Hence, the list has dichotomous values (applicable yes/no). Figure 1 in the Electronic Supplementary provides an overview of the complaints and their frequency distribution.

#### Medical data

Medical data was extracted from medical reports after having received patients’ consent. The data used in this study was mainly collected at time t0 and supplemented by data collected at t1, where additional information was available, e.g., on the implementation of the chemotherapy treatment. We used the following medical data as potential factors influencing the social participation at t3: mastectomy (yes versus no), tumor status (3 versus 1 to 2), affected lymph node (at least 1 node affected vs. none), chemotherapy at t0/t1 including neoadjuvant treatment (yes vs. no) and grading status (1 to 3). The grading status 1 to 3 describes in how far cancer cells have degenerated and how well they differentiate from normal breast cells. The following classifications apply: grade 1: well differentiated, grade 2: moderately differentiated and grade 3: poorly differentiated, i.e. the cancer cells look very different from normal cells and are likely to grow and spread faster. In addition, it was considered whether patients received hormone therapy at t3.

#### Social participation

Social participation was measured by questionnaire on 20 different leisure activities that was taken from the Socio-Economic Panel Study (GSOEP V.31). For each activity, participants were asked how often they do it in their leisure time. The answer categories were as follows: 1. daily, 2. at least once a week, 3. at least once a month, 4. less often, and 5. never. According to the definition by Levasseur et al. [10], social participation is “a person’s involvement in social activities that provide social interactions within his/her community or society” [23]. Hence, we excluded those activities that are mainly carried out alone (e.g., reading books or newspapers, repairs to the house, flat or vehicles or gardening). A principal component analysis (PCA) was carried out for the remaining variables in order to identify different dimensions of social participation. This analysis resulted in twelve social activities, which could be assigned to five dimensions of social participation: 1. socio-cultural participation, 2. social participation in institutions, 3. social participation in the private sphere, 4. social participation via social media, and 5. passive leisure time activities (Electronic Supplementary Material Table 1). We defined levels of socio-cultural participation and social participation in institutions as “low” if none of these activities were carried out at least monthly. Low levels of social participation in the private sphere were defined as reciprocal visits taking place less than once a week. With respect to social participation via social media, we defined participation as “low” if online social media were used less than daily. We chose this threshold as the majority of patients use social media daily. The use of computer, online, console, or smartphone games was not included in the calculation of the scale “social participation via social media,” as these activities are not considered as social participation supporting the HRQoL. Therefore, this scale only consists of the variable “use of social media or social networks.”

### Statistical analyses

First, we performed a principal component analysis (PCA) in order to identify different dimensions of social participation. The PCA method, closely related to factor analysis, is a technique for reducing the dimensionality of big datasets, increasing interpretability but at the same time minimizing information loss [24]. The PCA was conducted using fourteen items that measure various aspects of social participation. Our analysis, carried out with the command “varimax rotation,” revealed five factors with an eigenvalue of greater than 1.0, which is the typical value, according to Kaiser’s criterion, for accepting a factor. These five factors explain 53.9% of the total variance. Two variables were excluded due to factor loadings below 0.50. We have not included “passive leisure activities” in the further analysis, as this dimension only includes the item “doing nothing, hanging out and daydreaming,” which we do not consider to be representing social participation. Electronic Supplementary Material Table 1 displays the different dimensions of social participation and their factor loadings. The PCA was run using SPSS for windows (Version 29).

Differences in social participation activities between breast cancer patients and the general population of women were analyzed by means of logistic regression analyses. Social participation serves as the dependent variable and the sample-affiliation (patients versus general population) as the independent variable, with the general population (controls) being the reference group. We estimated predicted probabilities with the post estimation command “margins” that give adjusted prevalence to visualize the results. In addition, prevalence ratios (PR) were calculated as the effect size of the differences between patients and controls. We determined the PR based on the adjusted prevalence and calculated prevalence ratios using the STATA command “nlcom” [25]. To determine the statistical significance of the results, we calculated chi-square test and the 95% confidence intervals of the PR (95% CI). Results are significant if the confidence intervals do not contain the value 1, which means that the adjusted prevalence of the respective group deviates sufficiently from the reference category. The same statistical technique was used to analyze the influence of diagnosis-related complaints and medical data on social participation. All analyses were adjusted for age and education as potential confounding factors.

## Results

### Social participation of breast cancer patients compared to the general population

On average 5 years after primary surgery (t3), patients participate less in socio-cultural activities compared to women of the parallelized general population (Fig. 1). For example, 56.9% of patients but 64.9% of the women in the general population (controls) visit cafes, pubs, or restaurants at least monthly. In addition, 9.7% of patients but 15.7% of the controls visit operas, classical concerts, theatres and exhibitions at least monthly. Differences for these social activities reached statistical significance between patients and controls, as the confidence intervals did not include the value 1 (Electronic Supplementary Material Fig. 2). With respect to “social participation in institutions,” significant differences between patients and controls were found for citizens’ initiatives or voluntary association activities, with patients being less involved. With regard to the weekly “social participation in the private sphere” which refers to reciprocal visits by neighbors, friends, family members, and relatives, only small differences between patients and controls were observed. Regarding “social participation via social media,” patients use online social networks and chat services significantly more frequently on a daily basis (79.4%) than controls (72.0%). By contrast, compared to controls, patients tended to be less engaged in computer-, online-, console-, or smartphone-games.

**Fig. 2:**
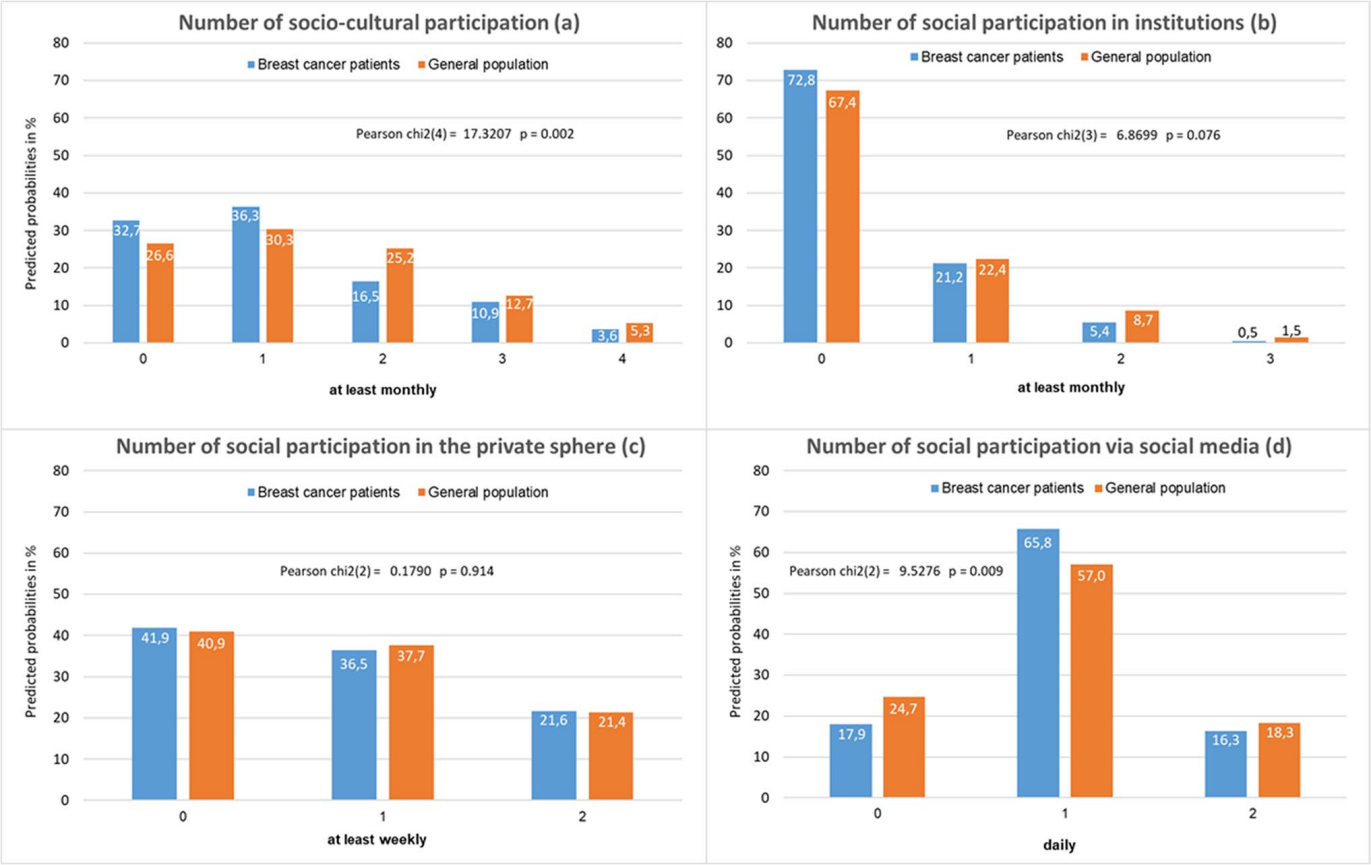
Number of social activities in four dimensions of social participation (**a** to **d**) among breast cancer patients (*n* = 346) as compared to women of the general population (*n* = 1035), in percentages

Considering the number of activities, it revealed for “socio-cultural participation” and “participation in institutions” that patients compared to controls are more likely to engage in none or only one activity while they are less likely to engage in two and more activities (Fig. 2). In contrast, there are hardly any differences between groups in the number of activities for “social participation in the private sphere.” With respect to “participation via social media,” patients are less likely to show no activity compared to controls.

### Associations between breast cancer-related complaints and patients’ social participation

**Fig. 3:**
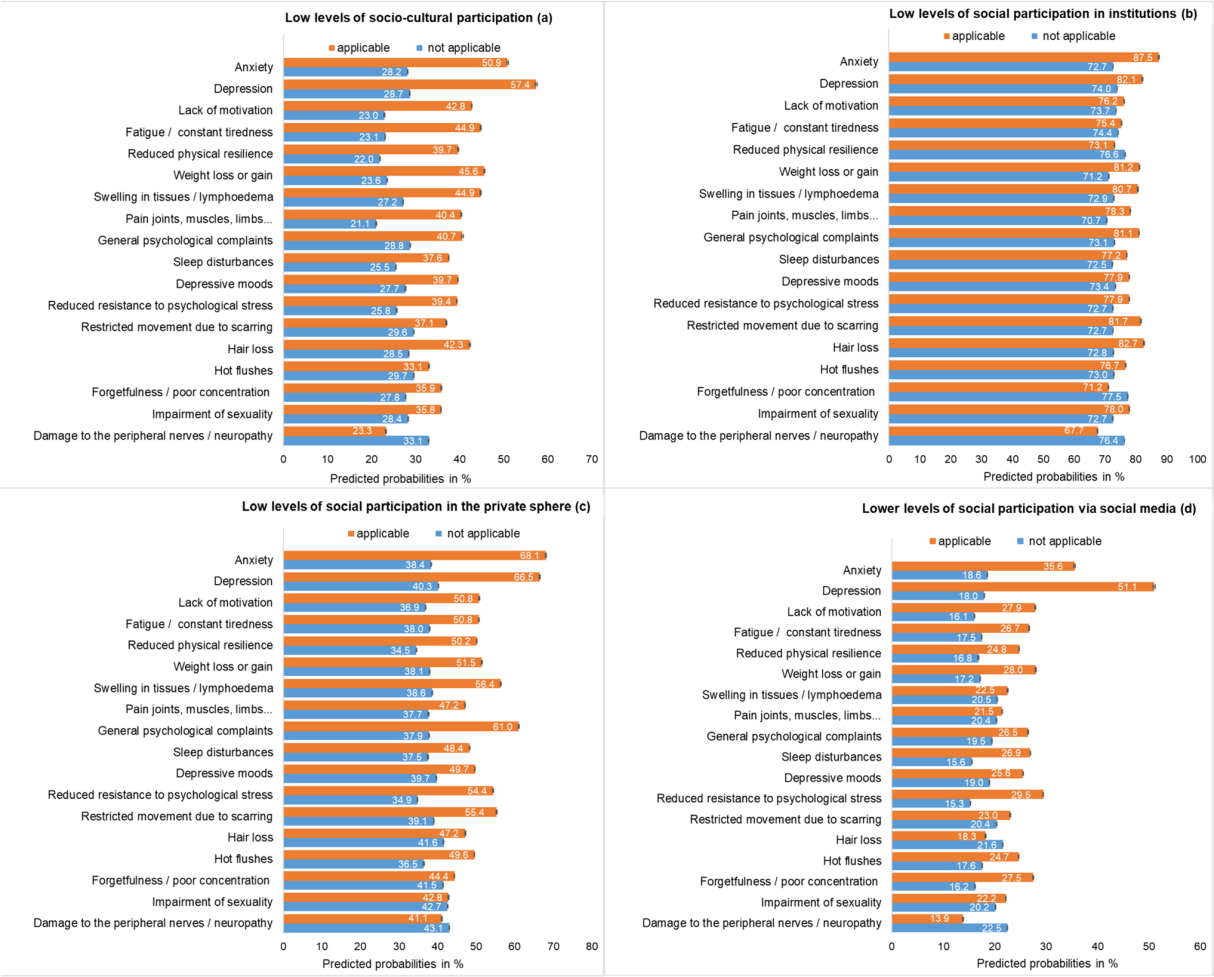
Adjusted prevalence (predicted probabilities) of low levels of social participation in four dimensions (**a** to **d**) among breast cancer patients (*n* = 346), stratified by diagnosis-related complaints

The frequencies of the diagnoses-related complaints in breast cancer patients at t3 ranged from 9.6% (depression) to 53.3% (pain in joints, muscles, limbs and others) (Electronic Supplementary Material Fig. 1). The association between these complaints and low levels of social participation was most pronounced for “socio-cultural participation” and “social participation in the private sphere” (Fig. 3). Patients with low levels of socio-cultural participation were significantly more likely to experience anxiety, depression, lack of motivation, fatigue, reduced physical resilience, weight loss or gain, and pain in the joints, muscles, and limbs (Electronic Supplementary Material Fig. 3). For example, of the patients with low levels of socio-cultural participation, 50.9% reported anxiety, compared to 28.2% of those with higher levels of socio-cultural participation (Fig. 3). In addition, patients with low levels of social participation in the private sphere are significantly more likely to have anxiety, depression, reduced physical resilience, tissue swelling, general psychological complaints, reduced mental resilience, and restricted mobility due to scarring (Electronic Supplementary Material Fig. 3). Patients with low levels of social participation in institutions only showed a significant higher frequency of anxiety, while those with low levels of social participation via social media showed significantly more frequent depression and reduced resistance to psychological stress.

### Associations between medical data and patients’ social participation

**Fig. 4:**
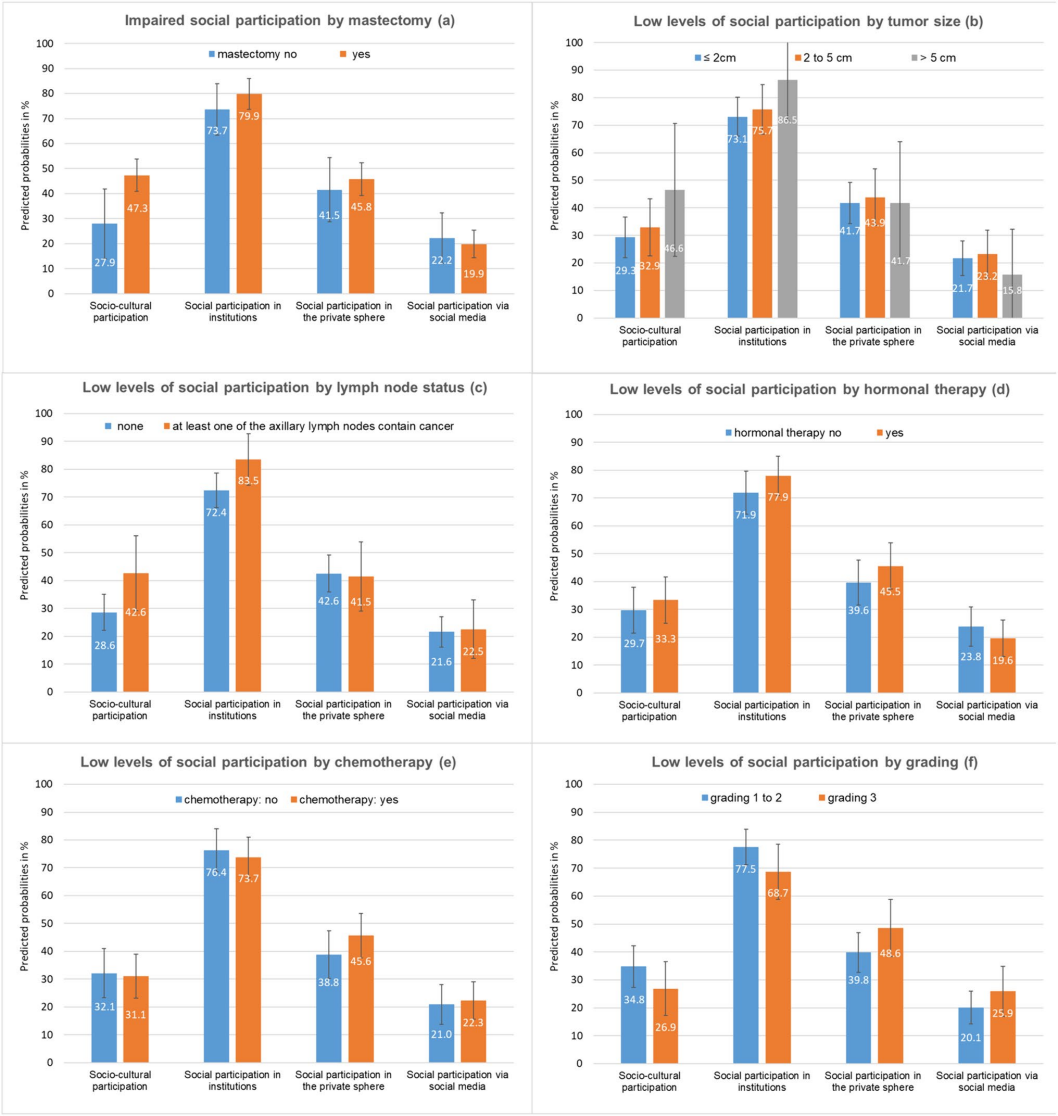
Adjusted prevalence (predicted probabilities) of low levels of social participation in four dimensions (**a** to **f**) among breast cancer patients (*n* = 346) stratified by medical data obtained at t0 and hormonal therapy at t3

Patients with a history of mastectomy were more likely to have low levels of social participation than patients who had undergone breast-conserving surgery (Fig. 4). A statistical significant effect of mastectomy was found for socio-cultural participation (Electronic Supplementary Material Fig. 4). Patients with worse outcomes at t0 (tumor status 3 and at least one affected lymph node) and those, who still received hormonal therapy at t3, tended to show lower levels of social participation, in particular with respect to social-cultural participation. However, these associations failed to reach statistical significance. Associations between chemotherapy-status or grading and social participation were inconsistent while the association with social media pointed in the opposite direction, indicating that patients with worse medical outcomes were not less but more likely to use social media daily. Again, these associations failed to reach statistical significance.

## Discussion

### Social participation in breast cancer patients compared to the general population

Our study revealed that on average 5 years after primary surgery, breast cancer patients show lower levels of social participation in the outdoor environment compared to women of the parallelized general population. In particular, this holds for socio-cultural participation, but also for citizens’ initiatives or voluntary association activities. Our findings indicate that the central goal of full re-adaption to society and of full participation in social life could not be confirmed in our study. As the concept of social participation is broadly formulated and lacks conceptual clarity [26], it is difficult to compare our results directly with the results of previous studies. However, previous research points in the same direction of impaired social participation in breast cancer patients. The study by Nikolić et al. found the lowest levels of patients’ satisfaction in the domains of recreation, communication and interpersonal relationships. The authors concluded that breast cancer patients had greater difficulty in maintaining their social activities than their everyday activities like nutrition, mobility or personal care [4]. The study by Ness et al. showed that cancer survivors have significantly more participation restrictions than people with no cancer history, for example in attending social events and outdoor events. These restrictions were still present many years following cancer diagnosis [27]. In addition, the study by Zhu et al. revealed that the social participation status improved 6 months after surgery; however, significant restrictions remained, particularly in social dimensions including social withdrawal and collective activities [11].

In contrast to social participation in the outdoor environment, we found no significant differences between patients and controls with respect to social participation in the private sphere that relates to indoor activities such as reciprocal visits by neighbors, friends, or family members. This indicates that activities taking place at home might not be affected by consequences of the breast cancer diagnosis and possible side effects of its treatment. As Hashidate pointed out, social participation can also be carried out through social networks by computer and mobile phone [23]. Regarding this kind of social participation, we found that breast cancer patients use online social networks and chat services significantly more frequent on a daily basis than controls. One interpretation of this finding might be that less social participation in outdoor activities might be compensated by higher use of social media. In addition, the higher use might be explained by the fact that it could be particularly important for them to receive disease-related information and support. Given that over 70% of patients in this study use social media on a daily basis, this resource could be utilized more in future for support structures.

### Influence of breast cancer-related complaints and medical data on patients’ social participation

Corresponding to previous findings [17, 21], we found diagnosis-related complaints to be frequently reported by the patients, even four to six years following primary surgery. As Nakamura et al. pointed out, many patients with breast cancer experience depression and anxiety after completing chemotherapy, which negatively affect their social activities and quality of life [28, 29]. In line with this, we found that in particular patients with low levels of socio-cultural participation were significantly more likely to experience pain and psychological symptoms including anxiety, depression, lack of motivation and fatigue. As oncologists often underestimate their patients’ depression and anxiety, Nakamura et al. recommend considering the inclusion of patient-reported outcomes to improve the monitoring of psychological symptoms after completing chemotherapy [28]. Our findings support this recommendation, as it may help to remove barriers that prevent patients from social activities. In addition, cognitive symptoms such as memory or attention difficulties may pose obstacles to engage in social activities [30]. Supporting this assumption, we found that women with complaints related to forgetfulness and poor concentration tended to be more affected by low levels of socio-cultural participation. Moreover, we found that patients with a worse medical outcome in terms of a history of mastectomy, a higher tumor status, at least one affected lymph node, and still receiving hormonal therapy at t3, tended to be affected more by low levels of social participation. A statistical significant result was found for the association between mastectomy and impaired socio-cultural participation. This corresponds with previous work by Zhu et al., who found that surgery type was a main factor influencing social participation. The authors argue that patients who underwent mastectomy generally had more challenging conditions and psychological burden which might explain lower levels of social participation [11]. Several studies have underlined the detrimental effects of an impaired body image following mastectomy on patients’ quality of life [31–33], which can in part be mitigated by reconstructing surgery of the breast [34]. Still, our findings indicate that women with mastectomies remain a vulnerable group who may have a greater need for support to carry out social activities in everyday life. In addition, Izydorczyk et al. point to the relevance of time getting on after surgery. They argue that the longer patients’ mastectomy dates back, the stronger their psychosocial response may be, characterized by a growing emotional instability, a sense of lost physical attractiveness, compromised self-esteem, a lack of control over one’s own life, as well as symptoms of depression and anxiety [32]. Social support delivered by family and friends and also by self-help-groups could be considered as one key factor in helping women after mastectomy to improve their psychological and physiological functioning and to restore their optimism [31]. Equally important, healthcare professionals can support the process of women’s self-image recovery after mastectomy through psychological counseling or physical exercises that strengthen their resilience and self-esteem [32]. In this context, it is also important to encourage communication about sexuality as an important part of patients' quality of life [35].

### Strengths and limitations

To the best of our knowledge, this is the first longitudinal study to explore levels of social participation of patients with breast cancer on average 5 years after surgery in Germany as compared to the general population of women, taking into account breast cancer-related complaints and medical data. The strengths of this study include the longitudinal and multicenter study design and the high response rate of 82.2% in relation to the initial sample (t0). However, we also acknowledged several study limitations. First, the study population based on breast cancer patients who were employed at t0. Thus, our findings cannot claim to be representative for all breast cancer patients. In addition, we used the GSOEP-data from the 2019 survey year, which we considered to be most comparable to the timing of our last survey wave, mainly conducted in 2023, when there were hardly any restrictions of the coronavirus pandemic left in Germany. However, we could not completely rule out the possibility that the different time references may have affected the results obtained.

## Conclusion

Compared to women in the general population, especially the socio-cultural participation of patients is still lower 5 years following first surgery. Our findings suggest that some breast cancer patients are prevented from participating fully in society by disease-related pain and psychological problems, emphasizing the need for interventions to support reintegration into society.

## Supplementary Information

Below is the link to the electronic supplementary material.

Electronic Supplementary Material Table 1 (The dimensions of social participation and their factor loadings – results of the Principal Component Analysis (PCA) DOCX 19.3 KB)

Electronic Supplementary Material Figure 1 (Frequencies of diagnosis-related complaints in breast cancer patients at t3 (*n* = 346) PNG 430 KB) (TIF 854 KB)

Electronic Supplementary Material Figure 2 (Prevalence Ratios (PRs) for social participation in four dimensions among breast cancer patients at t3 (*n* = 346) as compared to women of the general population (*n* = 1035) (TIF 1.09 MB)

Electronic Supplementary Material Figure 3 (Prevalence Ratios (PRs) for the effect of diagnosis-related complaints on low levels of social participation in four dimensions at t3 (a to d) (TIF 980 KB)

Electronic Supplementary Material Figure 4 (Prevalence Ratios (PRs) for the effect of medical data obtained at t0 and hormonal therapy at t3 on low levels of social participation in four dimensions at t3 (a to f) (TIF 766 KB)

## Data Availability

The data are available from the authors upon reasonable request.
