# Supplementary material for: Social participation of women with breast cancer compared to the general population 5 years after primary surgery—what role do medical data and cancer-related complaints play?
Source: Support Care Cancer. 2024 Aug 2;32(8):566. doi: 10.1007/s00520-024-08695-w (PMC11297071; doi:10.1007/s00520-024-08695-w)
Supplement: Supplementary file 1 — (The dimensions of social participation and their factor loadings – results of the Principal Component Analysis (PCA) DOCX 19.3 KB) [file 520_2024_8695_MOESM1_ESM.docx]

**Appendix Tab. 1:** The dimensions of social participation and their factor loadings – results of the Principal Component Analysis (PCA)

|  | **Factor 1**  **Socio-cultural participation** | **Factor 2**  **Social participation in institutions** | **Factor 3**  **Social participation in the private sphere** | **Factor 4**  **Social participation via social media** | **Factor 5**  **Passive leisure time activities** |
| --- | --- | --- | --- | --- | --- |
| Visits to cafes, pubs and restaurants | .647 |  |  |  |  |
| Visits to opera, theatre, classical concerts and exhibtions | .635 |  |  |  |  |
| Visits to cinema, pop and jazz concerts and clubs | .729 |  |  |  |  |
| Excursions and short trips | .654 |  |  |  |  |
| Participation in political parties, local politics and citizens' initiatives |  | .516 |  |  |  |
| Voluntary activities in clubs and associations, social services |  | .760 |  |  |  |
| Church visits and visits to religious events |  | .737 |  |  |  |
| Reciprocal visits from neighbors, friends and acquaintances |  |  | .530 |  |  |
| Reciprocal visits from family members or relatives |  |  | .738 |  |  |
| Use of social media or social networks |  |  |  | .551 |  |
| Computer-, online-, console- or smartphone-games |  |  |  | .799 |  |
| Hanging out, dreaming |  |  |  |  | .953 |

Notes: Kaiser-Meyer-Olkin (KMO) measure of sampling adequacy = 0.76 (values less than 0.5 are unacceptable), Bartlett’s test of sphericity = p <.001 (rejection of the null hypothesis that variables are unrelated and not ideal for factor analysis). Displayed are the factor loadings of the rotated component matrix, factor values above 0.49 are displayed, 5 factors (eigenvalue above 1) explain 53.9% of the variance, of which factor 1 explains 20.1%, factor 2= 10.3%, factor 3 = 8.6%, factor 4 = 7.8% and factor 5 = 7.2%.
