# Supplementary figures and images for: Social participation of women with breast cancer compared to the general population 5 years after primary surgery—what role do medical data and cancer-related complaints play?
Source: Support Care Cancer. 2024 Aug 2;32(8):566. doi: 10.1007/s00520-024-08695-w (PMC11297071; doi:10.1007/s00520-024-08695-w)

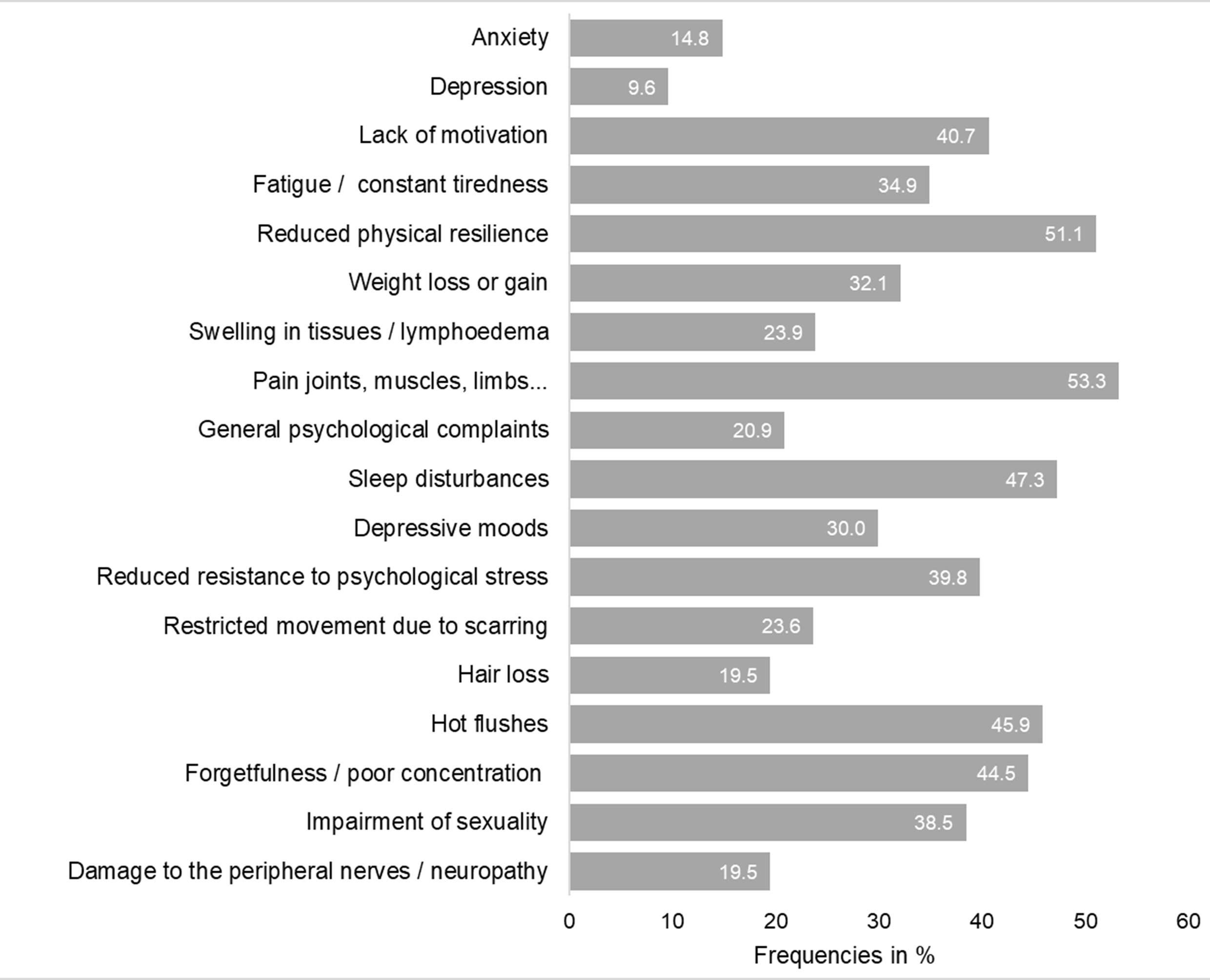

Supplement: Supplementary file 2 — (Frequencies of diagnosis-related complaints in breast cancer patients at t3 (n = 346) PNG 430 KB) (TIF 854 KB) [file 520_2024_8695_MOESM2_ESM.tif]

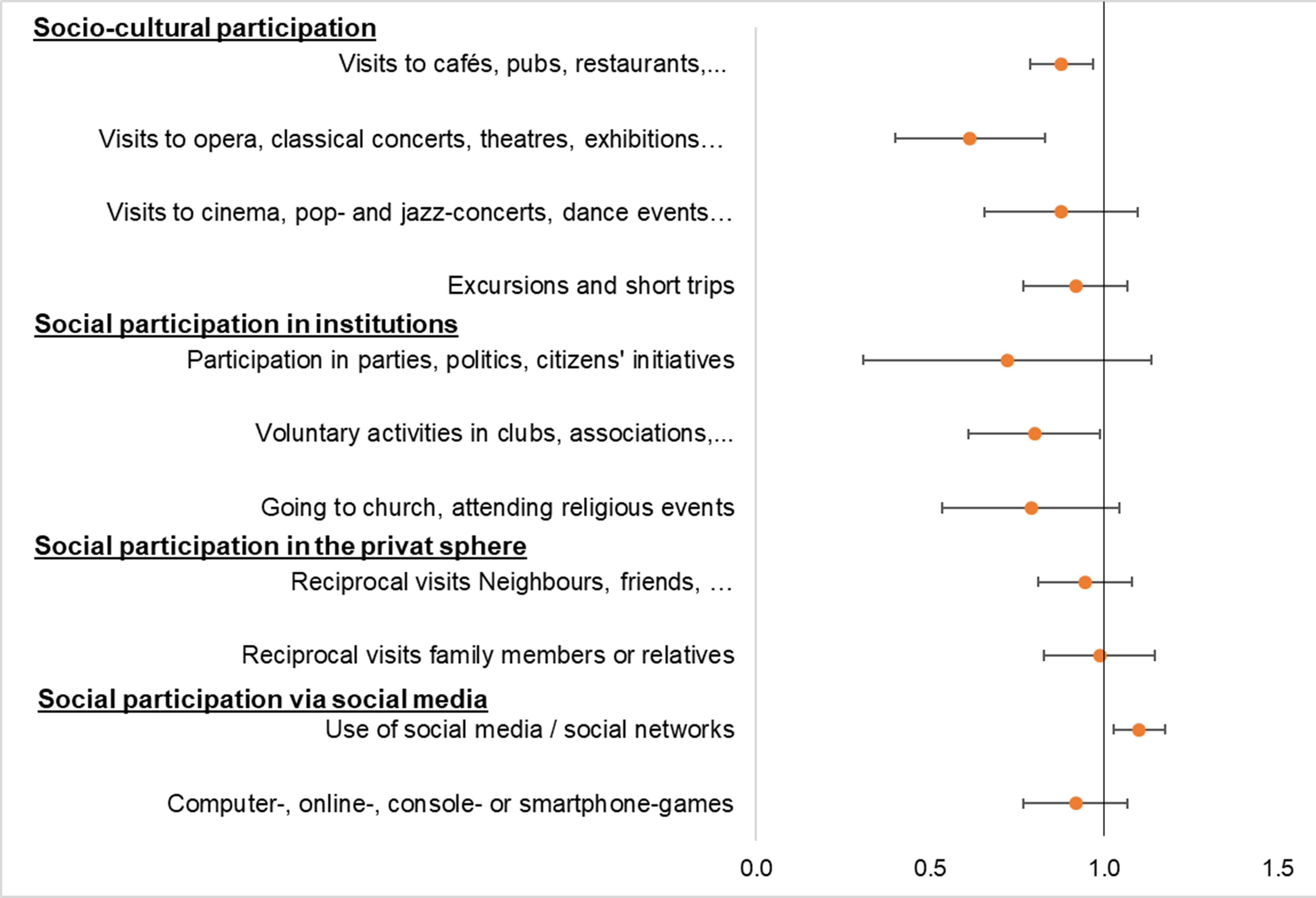

Supplement: Supplementary file 3 — (Prevalence Ratios (PRs) for social participation in four dimensions among breast cancer patients at t3 (n = 346) as compared to women of the general population (n = 1035) (TIF 1.09 MB) [file 520_2024_8695_MOESM3_ESM.tif]

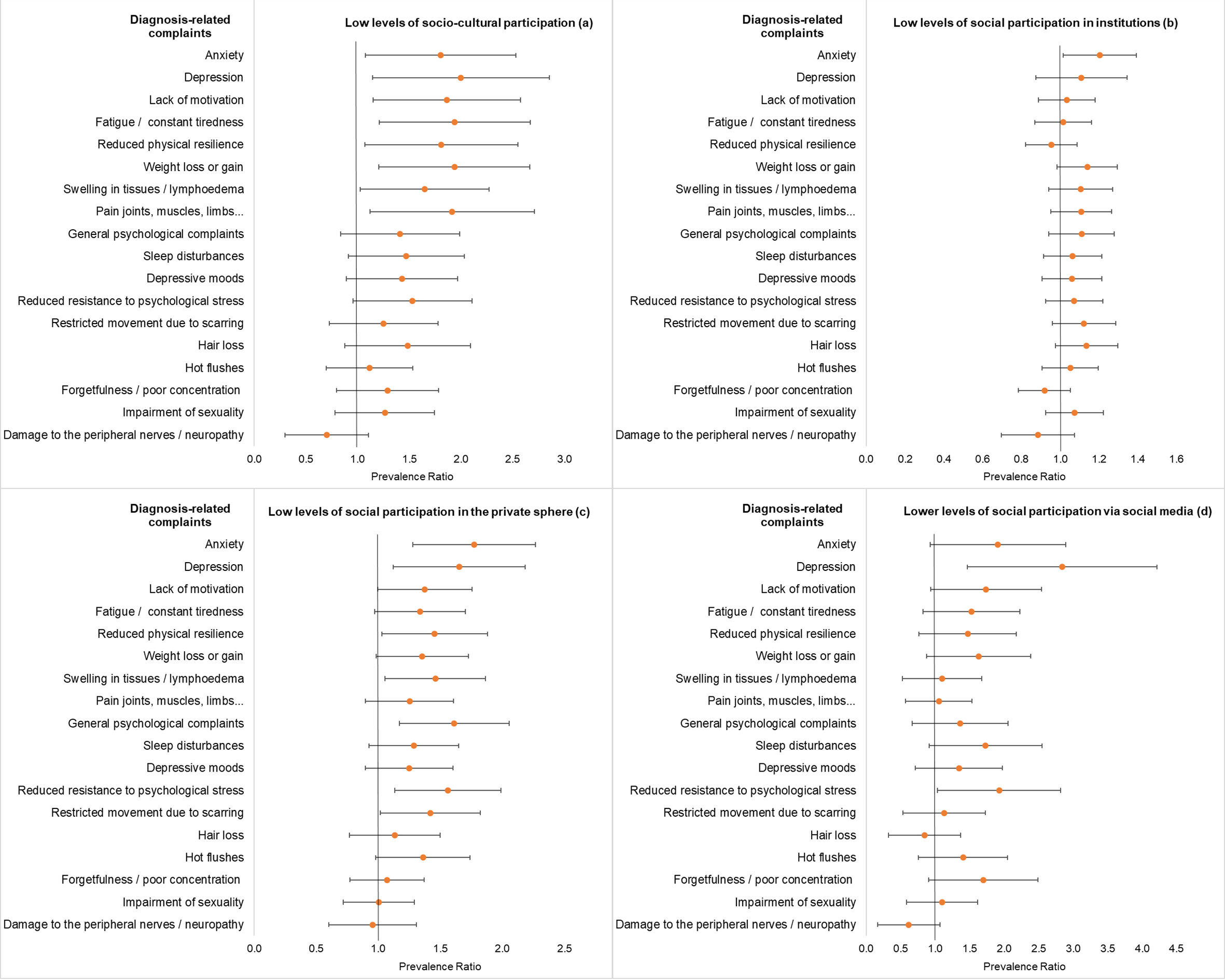

Supplement: Supplementary file 4 — (Prevalence Ratios (PRs) for the effect of diagnosis-related complaints on low levels of social participation in four dimensions at t3 (a to d) (TIF 980 KB) [file 520_2024_8695_MOESM4_ESM.tif]

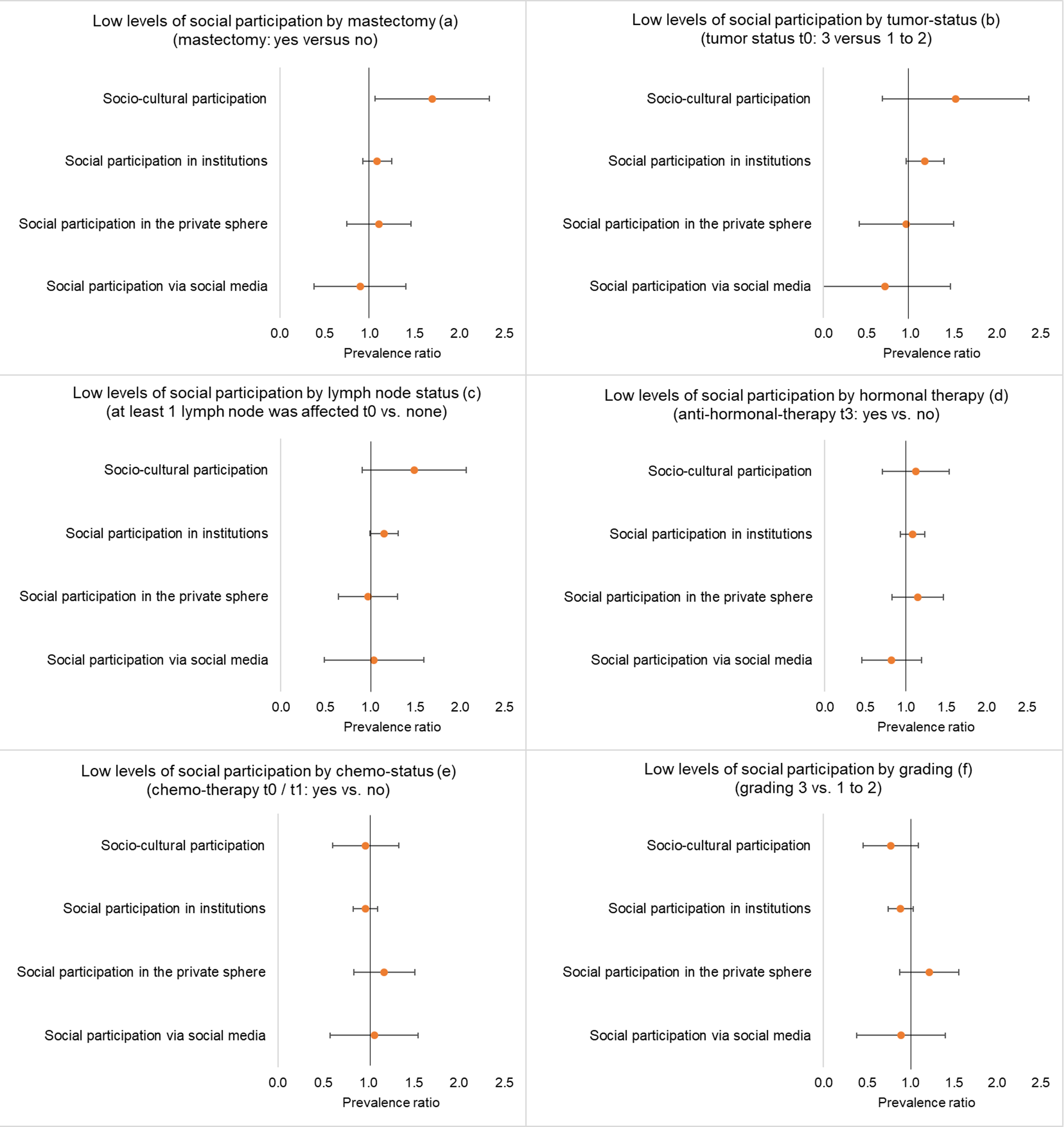

Supplement: Supplementary file 5 — (Prevalence Ratios (PRs) for the effect of medical data obtained at t0 and hormonal therapy at t3 on low levels of social participation in four dimensions at t3 (a to f) (TIF 766 KB) [file 520_2024_8695_MOESM5_ESM.tif]
